# Supplementary material for: Time-series transcriptome analysis identified differentially expressed genes in broiler chicken infected with mixed Eimeria species
Source: Front Genet. 2022 Aug 8;13:886781. doi: 10.3389/fgene.2022.886781 (PMC9393255; doi:10.3389/fgene.2022.886781)
Supplement: Supplementary file 2 [file DataSheet1.ZIP › 7dpi_GO.Gsea.1625106495923/GOBP_RETINAL_METABOLIC_PROCESS.html]

Details for gene set GOBP\_RETINAL\_METABOLIC\_PROCESS[GSEA]

|  || Dataset | TMM\_7dpi\_gct\_format\_7dpi\_gct\_format.Class\_4dpi.cls #PC\_versus\_NC.Class\_4dpi.cls #PC\_versus\_NC\_repos |
| Phenotype | Class\_4dpi.cls#PC\_versus\_NC\_repos |
| Upregulated in class | 0 |
| GeneSet | GOBP\_RETINAL\_METABOLIC\_PROCESS |
| Enrichment Score (ES) | -0.75304973 |
| Normalized Enrichment Score (NES) | -1.806988 |
| Nominal p-value | 5.2882073E-4 |
| FDR q-value | 0.12290614 |
| FWER p-Value | 0.9502 |
Table: GSEA Results Summary

  

Fig 1: Enrichment plot: GOBP\_RETINAL\_METABOLIC\_PROCESS      
 Profile of the Running ES Score & Positions of GeneSet Members on the Rank Ordered List

  

| SYMBOL | TITLE | RANK IN GENE LIST | RANK METRIC SCORE | RUNNING ES | CORE ENRICHMENT || 1 | DHRS13 | na | 1561 | 0.364 | -0.0791 | No |
| 2 | RDH11 | na | 2695 | 0.164 | -0.1506 | No |
| 3 | RDH10 | na | 2781 | 0.153 | -0.1363 | No |
| 4 | ALDH1A2 | na | 3486 | 0.064 | -0.1860 | No |
| 5 | SDR16C5 | na | 4584 | -0.059 | -0.2692 | No |
| 6 | BCO2 | na | 5258 | -0.127 | -0.3075 | No |
| 7 | BCO1 | na | 7142 | -0.324 | -0.4191 | No |
| 8 | RPE65 | na | 8487 | -0.482 | -0.4636 | No |
| 9 | ALDH1A3 | na | 11961 | -1.725 | -0.5117 | Yes |
| 10 | ALDH8A1 | na | 11963 | -1.728 | -0.2700 | Yes |
| 11 | CYP1B1 | na | 11987 | -1.957 | 0.0019 | Yes |
Table: GSEA details [plain text format]

  

Fig 2: GOBP\_RETINAL\_METABOLIC\_PROCESS      
 Blue-Pink O' Gram in the Space of the Analyzed GeneSet

  

Fig 3: GOBP\_RETINAL\_METABOLIC\_PROCESS: Random ES distribution      
 Gene set null distribution of ES for **GOBP\_RETINAL\_METABOLIC\_PROCESS**
